# Molecular Modeling of Protein Corona Formation and Its Interactions with Nanoparticles and Cell Membranes for Nanomedicine Applications

**DOI:** 10.3390/pharmaceutics13050637

**Published:** 2021-04-29

**Authors:** Hwankyu Lee

**Affiliations:** Department of Chemical Engineering, Dankook University, Yongin-si 16890, Korea; leeh@dankook.ac.kr

**Keywords:** protein corona, molecular dynamics simulation, drug delivery, nanomedicine, protein-nanoparticle interaction, protein-protein interaction

## Abstract

The conformations and surface properties of nanoparticles have been modified to improve the efficiency of drug delivery. However, when nanoparticles flow through the bloodstream, they interact with various plasma proteins, leading to the formation of protein layers on the nanoparticle surface, called protein corona. Experiments have shown that protein corona modulates nanoparticle size, shape, and surface properties and, thus, influence the aggregation of nanoparticles and their interactions with cell membranes, which can increases or decreases the delivery efficiency. To complement these experimental findings and understand atomic-level phenomena that cannot be captured by experiments, molecular dynamics (MD) simulations have been performed for the past decade. Here, we aim to review the critical role of MD simulations to understand (1) the conformation, binding site, and strength of plasma proteins that are adsorbed onto nanoparticle surfaces, (2) the competitive adsorption and desorption of plasma proteins on nanoparticle surfaces, and (3) the interactions between protein-coated nanoparticles and cell membranes. MD simulations have successfully predicted the competitive binding and conformation of protein corona and its effect on the nanoparticle–nanoparticle and nanoparticle–membrane interactions. In particular, simulations have uncovered the mechanism regarding the competitive adsorption and desorption of plasma proteins, which helps to explain the Vroman effect. Overall, these findings indicate that simulations can now provide predications in excellent agreement with experimental observations as well as atomic-scale insights into protein corona formation and interactions.

## 1. Introduction

**Figure 1:**
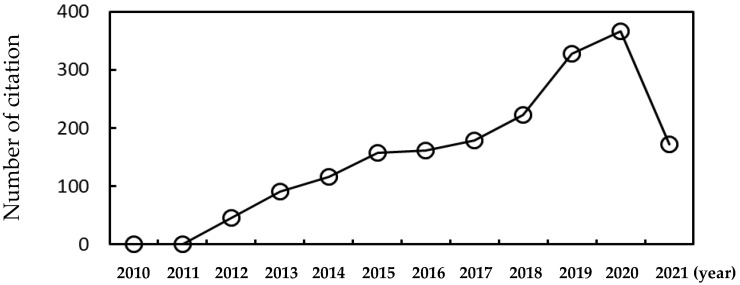
The number of citation of research articles under the topic of protein corona studied by molecular dynamics simulations as of April/2021.

Nanoparticles have been studied for drug delivery and antitumor therapeutics [1,2,3,4,5]. Experimental and theoretical studies have mostly focused on modulating the size, structure, and surface properties of nanoparticles, in order to increase the targeting efficiency of nanoparticles [6,7,8,9,10]. However, when nanoparticles flow through the bloodstream, they interact with various plasma proteins and, thus, their surfaces are often covered by multiple protein layers, a process called protein corona [11,12]. The formation of protein corona influences conformations and surface properties of nanoparticles, which can significantly increase or decrease the targeting efficiency and cytotoxicity via electrostatic and hydrophobic interactions between nanoparticles and cell membranes [11,13,14,15,16,17,18]. The mechanism of protein corona formation and the effect of protein corona on nanoparticle properties and interactions with cell membranes need to be studied at nearly the atomic scale in order to understand this and complement relevant experiments, as can be done using molecular dynamics (MD) simulations. Because the system containing multiple plasma proteins and nanoparticles is too large to simulate using all-atom models, theoretical studies, such as kinetic mean-field theory (MFT), density functional theory (DFT), and monte carlo (MC) simulations have been mainly performed until 2000s, but advances on computer power and force-field development make it possible to perform MD simulations of the nanoparticle complexed with plasma proteins for the past decade. Figure 1 shows that citations for research papers regarding MD simulations under the topic of protein corona have drastically increased, which will be thoroughly reviewed here, with a focus on plasma proteins, but not on other molecules, such as peptides and synthetic polymers. Zhdanov’s review paper is highly recommended for MFT, DFT, and MC simulations of protein corona on nanoparticles [19]. In this review, we aim to show that MD simulations can successfully predict the experimentally observed compositions and structures of protein corona and their interactions with lipid membranes, as. well as help to explain the mechanism of the Vroman effect regarding the competitive adsorption and desorption of plasma proteins on the nanoparticle surface.

## 2. Conformation, Binding Site and Strength of Plasma Proteins on the Nanoparticle

### 2.1. Gold Nanoparticles

**Figure 2:**
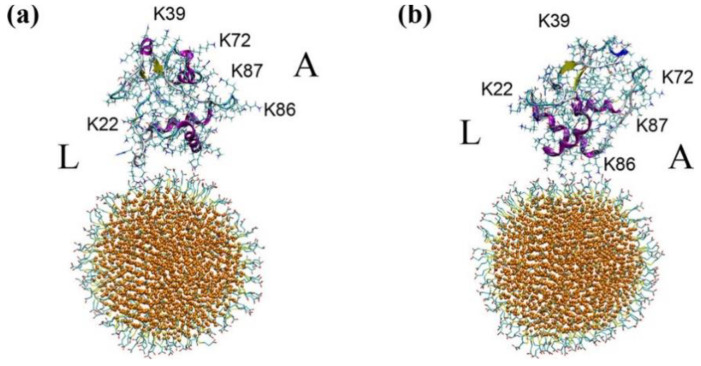
Orientations of cytochrome *c* adsorbed onto the 4 nm-sized mercaptopropionic acid-functionalized AuNP: (**a**) K22 and K86 facing the NP, (**b**) K86 and K87 facing the NP. Reproduced with permission from [26], American Chemical Society, 2019.

Wang et al. simulated bovine serum albumin (BSA) that was adsorbed onto gold nanorods (AuNRs) and determined the binding site and conformational change of BSA [20]. Ramezani and Rafii-Tabar simulated human serum albumin (HSA) that was adsorbed onto gold nanoparticles (AuNPs), which showed the unfolding of adsorbed HSA and determined the key amino acids for the HSA-AuNP binding [21]. Tavanti et al.’s coarse-grained (CG) simulations showed that, when ubiquitin binds to AuNP, the protein is reoriented on the AuNP surface to optimize the ubiquitin-AuNP interaction, depending on the particle size and environment [22]. Shao and Hall’s CG simulations showed that isotherms of protein adsorption are well described by the Langmuir, Freundlich, Temkin, and Kiselev models, which suggests a generalized model for the adsorption of proteins onto nanoparticles [23]. They also found that the HSA-AuNP binding induces the flexibility and structural changes of HSA, which allosterically influences the binding affinity of HSA to fatty acids, thyroxin, and metals [24]. Yang et al. simulated beta-lactoglobulin that was bound to AuNP and determined its binding site and structural change, which are interpreted by electrostatic interactions [25]. Tollefson et al. simulated cytochrome *c* that was bound to a mercaptopropionic acid-functionalized AuNP and determined the preferred binding orientations of adsorbed proteins (Figure 2) [26].

**Figure 3:**
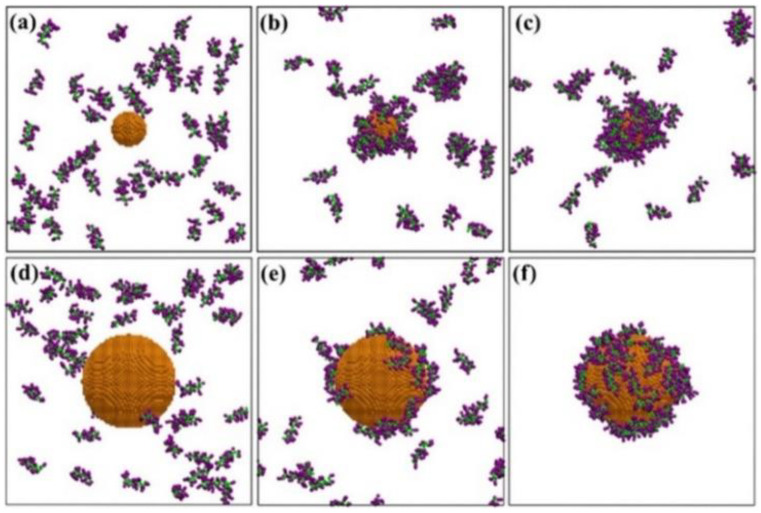
Snapshots of simulations of ovispirin corona adsorbed onto the surface of (**a**–**c**) 3.2 nm-and (**d**–**f**) 10 nm-sized gold nanoparticles. Reproduced with permission from [30], American Chemical Society, 2020.

Power et al. calculated the adsorption free energies for amino acids and various plasma proteins, such as surfactant protein, ubiquitin, hemoglobin, myoglobin, transferrin (TF), macroglobulin alpha, HSA, alpha-1-antitrypsin, and immunoglobulin A, E, and G (IgA, IgE, and IgG), which helps to predict their binding affinity to differently sized AuNPs [27]. Lu et al. simulated HSA, IgE, and apolipoprotein (APO) that were bound to graphene and Au nanosheets, showing that the binding strength and secondary structures of proteins can be modulated by the number and distribution of hydroxyl groups that were grafted on the nanosheet surface [28]. Taha and Lee performed DFT, MC, and MD simulations of the adsorption of cyclo-alanine dipeptide (c(AA)) molecules onto AuNP and compared the interaction forces of c(AA)-water, c(AA)-c(AA), and AuNP-c(AA), showing that methyl groups of alanine sidechains strengthen the binding between peptides and Au clusters [29]. Jahan Sajib et al. simulated ovispirin-1 and lysozyme adsorbed onto AuNP, showing that adsorbed ovispirin and lysozyme, respectively, form a homogeneous single layer and inhomogeneous multilayers on the AuNP surface, and that the size of AuNP influences the structural change and binding orientation of adsorbed proteins (Figure 3) [30]. 

### 2.2. Silver Nanoparticles

Kakinen et al. performed both all-atom and CG simulations of a luciferase molecule that was adsorbed onto the citrated-coated silver nanoparticle (AgNP) and observed its structural change upon binding to AgNP because of electrostatic interactions between anionic luciferase residues and cationic AgNP surfaces [31]. Ding et al. found that the adsorption amount of ubiquitin onto AgNP follows an unusual stretched-exponential binding kinetics instead of a typical single exponential binding kinetics, in agreement with the experiments [32]. Li et al. also observed that, when APO adsorbs to the AgNP surface, their dynamics and structures change, depending on the ion concentration [33]. Wang et al. performed replica-exchange discrete MD simulations of lysozyme and alpha-lactalbumin (ALact) interacting with AgNPs, showing different effects of protein adsorption on the secondary structure and binding strength, consistent with the experimental results [34]. Nayak et al. simulated bovine lactoferrin (BLf) adsorbed onto AgNP and found that the BLf-AgNP binding is modulated by van der Waals interactions and hydrogen bonds [35].

### 2.3. Carbon Nanomaterials

Ge et al. simulated bovine fibrinogen (FG), IgG, TF, and BSA adsorbed onto the single-walled carbon nanotube (SWCNT), showing that contact residue numbers and surface areas of adsorbed proteins are in the order of FG > IgG > TF > BSA, in agreement with experiments [36]. In particular, they found that π-π stacking interactions are important for the binding between proteins and SWCNTs [36]. Sengupta et al. simulated BSA that was interacting with differently charged carbon nanomaterials, showing the unfolding of BSA and enhanced conformational entropy [37]. Our group simulated HSA and FG interacting with SWCNTs covalently functionalized or noncovalently coated with polyethylene glycol (PEG) chains of different sizes and grafting densities, showing that spherical HSA more weakly bind to the SWCNT than the linearly-shaped FG do, and that PEG chains can sterically suppress the adsorption of plasma proteins onto the SWCNT surface [38].

### 2.4. Polymer-Grafted Nanoparticles

**Figure 4:**
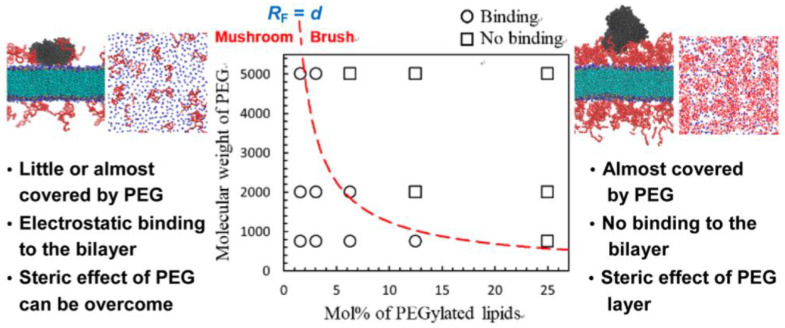
The adsorption of human serum albumin (HSA; black color) onto the PEGylated membrane surface (PEG in red), depending on mushroom and extended-brush conformations of PEG chains with different sizes and grafting densities. Reproduced with permission from [39], American Chemical Society, 2016.

Our group performed CG simulations of HSA interacting with PEG-grafted membranes, showing that the adsorption of HSA onto the membrane surface is sterically suppressed by grafted PEG chains in an extended brush, but not in a mushroom (Figure 4), in agreement with experiments showing less adsorption of plasma proteins onto the nanoparticle or liposome surface that was grafted with PEG in the brush state [39]. Settanni et al. determined the composition of specific amino acids that bind to PEG- and poly(phosphoester)-coated nanoparticles [40]. They also simulated the adsorption of HSA onto the nanoparticle that was coated with hydrophilic polymers and analyzed the kinetics of protein adsorption and its effect on the polymer conformation [41].

### 2.5. Others

**Figure 5:**
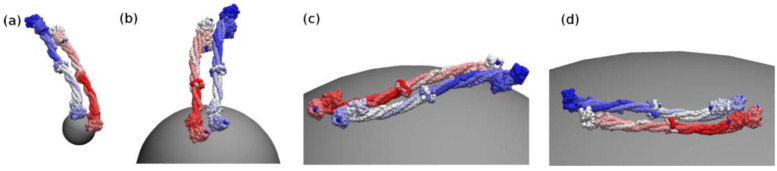
Orientations of fibrinogen (FG) adsorbed onto a neutral nanoparticle of radius (**a**) 5, (**b**) 20, (**c**) 50, and (**d**) 100 nm. Reproduced with permission from [42], American Institute of Physics, 2015.

Lopez and Lobaskin performed CG simulations of HSA, antitrypsin, macroglobulin, FG, TF, and IgG that were bound to charged and neutral nanoparticles of different sizes, showing the dependence of the binding affinity on the protein type, particle size and electrostatics (Figure 5) [42]. In particular, they ranked the binding affinity of those six plasma proteins, in agreement with the experimental results [42,43].

**Figure 6:**
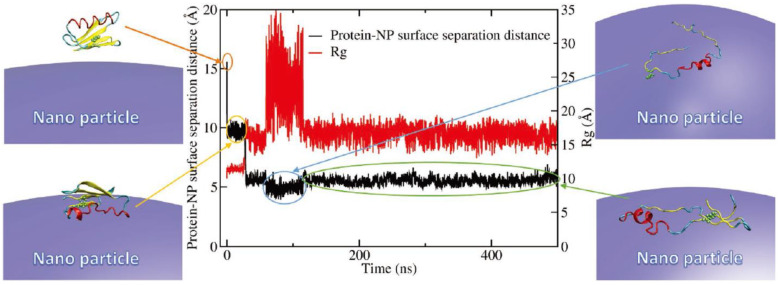
The distance between a protein and a nanoparticle surface, and the radius of gyration of a protein as a function of simulation time. Reproduced with permission from [45], WILEY-VCH, 2017.

Yu and Zhou simulated the adsorption of lysozyme onto differently sized silica-nanoparticles, showing the effect of particle curvature on the interfacial hydration, orientation, and conformational change of adsorbed proteins [44]. Wei et al. developed a CG model that can be applied to predict the conformation and adsorption behavior of proteins that were adsorbed onto nanoparticles with different sizes and surface groups (Figure 6), in agreement with experimental results [45].

**Figure 7:**
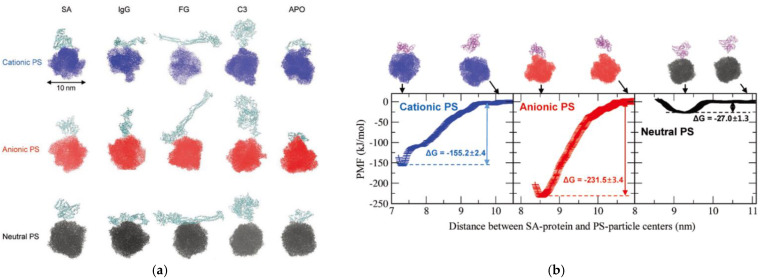
(**a**) Final snapshot of simulations of the binding between differently charged polystyrene (PS) nanoparticles and major plasma proteins such as HSA, immunoglobulin G (IgG), FG, complement 3 (C3), and apolipoprotein (APO), (**b**) calculations of potentials of mean force (PMF; binding free energies) between HSA and differently charged PS nanoparticles. Reproduced with permission from [57], WILEY-VCH, 2020.

Pilkington et al. observed the effect of lysozyme and aLact adsorption on the amyloid-polypeptide aggregation, fibril formation, and cytotoxicity [46]. Wang et al. simulated BSA that was bound to L-chiral and D-chiral surfaces, showing that the orientation, binding site, and strength depend on the chiral type of surfaces [47]. Tavakol et al. performed all-atom and CG simulations of FG interacting with polystyrene (PS) nanoparticles in the presence of model metabolites of diabetes and hypercholesterolemia, showing that the presence of glucose and cholesterol influences the binding site and density of adsorbed FG [48]. Wang et al. simulated the adsorption of HSA and IgE onto polyamidoamine dendrimers and found that the modification of dendrimer surfaces with neutral chains suppresses the binding between charged proteins and dendrimer surfaces [49]. Fardanesh et al. observed the folded structure of proteins upon binding to the TiO_2_-nanoparticle cluster [50]. Moya et al. simulated multiple HSA molecules that were bound to the iron-oxide nanoparticle surface and calculated the thickness of protein layers on differently sized nanoparticles, in agreement with the experimental results [51]. Derakhshankhah et al.’s simulations showed the non-cooperative binding between FG and zeolite nanoparticles via hydrogen bonds and electrostatic interactions of D-domain of FG [52]. Xu and Dzubiella calculated the binding free energies of lysozyme that was adsorbed onto the highly charged dendritic polyglycerol sulfate and derived the concept of a coverage-dependent binding affinity in the Langmuir model [53]. Sanchez-Guzman et al. simulated oxyhemoglobin interacting with silica surfaces at pH 7 and pH 9 under different temperatures of 295 K, 322 K, 353 K, and 400 K, showing the dependence of the binding strength and structure on temperature and pH conditions [54]. Qi et al. simulated TF that was bound to various cadmium selenide (CdSe) surfaces, such as (100) and (002) facets, and found that disulfide moieties of TF interact with the CdSe (100) surface rather than with the CdSe (002) surface, indicating the effect of different facets on the binding strength [55]. Hassanian et al. showed that HSA binds to zinc oxide nanoparticles (ZnO NPs) mainly via electrostatic interactions between charged groups of HSA and ZnO NP, leading to the structural change of adsorbed HSA [56]. Our group calculated the binding free energies between differently charged PS particles and five major plasma proteins, such as HSA, IgG, FG, complement C3 (C3), and APO, showing that all of the simulated plasma proteins bind to anionic, cationic, and neutral PS particles, although their binding strengths are higher for charged PS particles than for neutral PS particles (Figure 7) [57].

These MD simulations have predicted the conformational change, binding site, and strength of plasma proteins that were bound to the nanoparticle surface. However, nanoparticles have diverse structural and surface properties and, hence, these results should be applied to differently sized, shaped, and functionalized nanoparticles with caution. More modeling efforts are still ongoing to develop a generalized model to predict the protein-nanoparticle binding in terms of their size, shape, structure, and surface properties, such as electrostatics and hydrophobicity.

## 3. Competitive Adsorption and Desorption of Plasma Proteins on Nanoparticle Surfaces

**Figure 8:**
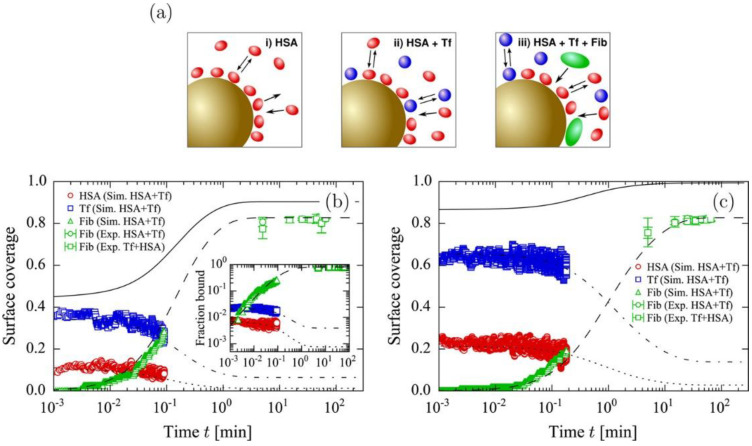
Adsorption of FG (green) on silica NPs precoated with HSA (red) first and TF (blue) next. (**a**) Schematic illustration of three steps for the adsorption process. (**b**) Surface coverage of HSA, TF, and FG adsorbed onto silica NPs as a function of time. (**c**) Surface coverage of HSA, TF, and FG adsorbed onto silica NPs at high protein concentrations. Reproduced with permission from [60], American Chemical Society, 2016.

Experimental and theoretical studies have shown that, when plasma proteins adsorb to the nanoparticle and form protein layers that are composed of hard (inner) and soft (outer) corona, abundant plasma proteins first bind to the nanoparticle surface, and they they are then replaced by high-affinity proteins via the adsorption competition between proteins, called the Vroman effect [58], although this mechanism has not been well interpreted at the atomic level. To resolve this, Vilaseca et al. developed CG models of HSA, FG, and IgG as simple shapes, and showed that small (HSA) and large (FG) proteins do not compete with each other, but rather compete with others (IgG) in a cooperative way, which supports the Vroman effect [59]. Vilanova et al. also developed CG models for protein-silica NP and protein–protein interactions, showing that three-body interactions can be applied to capture the kinetics of the competitive adsorption of plasma proteins, such as HSA, TF, and FG, which successfully reproduces the experimental observation regarding the replacement of abundant proteins (HSA and TF) with high-affinity proteins (FG) at the early stage of corona formation (Figure 8) [60]. In particular, the adsorption kinetics and compositions of HSA, TF, and FG agree with the experimental results, showing the ability of the CG model (three-body interaction) to predict the kinetics and composition of protein corona on nanoparticle surfaces [60].

**Figure 9:**
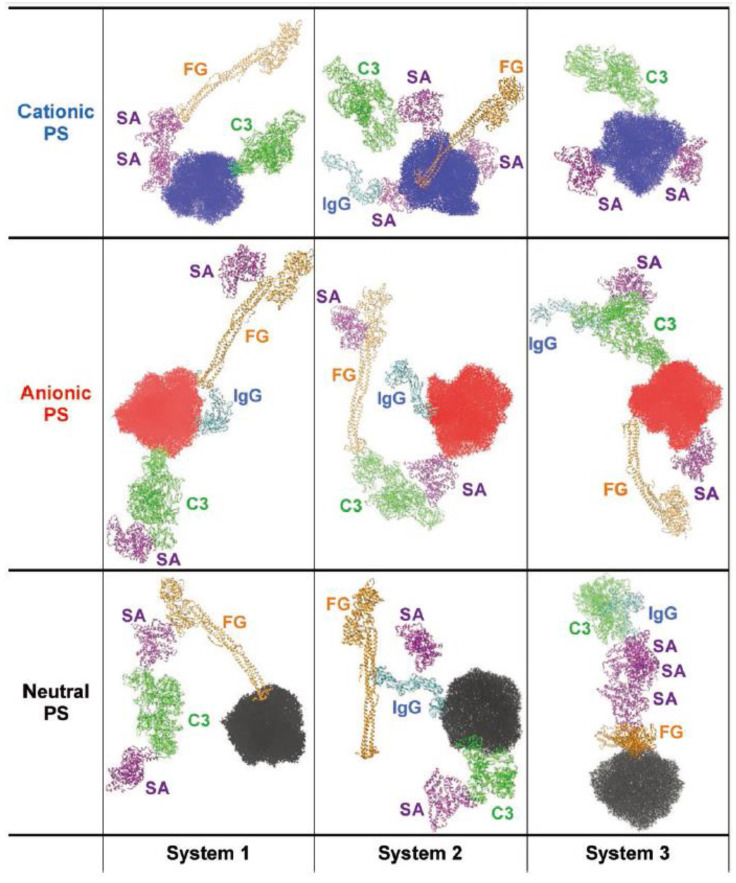
Final snapshots of simulations showing the self-assembly of plasma proteins randomly adsorbed onto cationic, anionic, and neutral PS nanoparticles. Reproduced with permission from [57], WILEY-VCH, 2020.

Tavanti et al. performed CG simulations of multiple insulin and FG molecules that were adsorbed onto 5 nm-sized citrate-capped AuNPs, and showed that 20 insulin and three FG molecules bind to the AuNP surface, indicating the difference of their binding affinity [61]. In particular, insulin has the specific binding site, while FG has various binding sites, depending on the protein concentration and composition [61]. The presence of FG induces less adsorption of insulin, showing the adsorption competition between insulin and FG [61]. They also simulated multiple hemoglobin, myoglobin, and trypsin molecules that were adsorbed to the 15 nm-sized citrate-capped AuNP, showing that the competition between proteins influences the final composition of protein corona on AuNP [62]. These CG simulations revealed the competition between simple model proteins and their adsorption kinetics, although atomic-level mechanisms of protein–protein and protein–NP interactions have not been well understood. In fact, CG models do not have an ability to reproduce the structural change of proteins, which may influence protein–protein and protein–NP interactions. To overcome these limitations of CG models and understand the atomic-level interactions, our group recently performed all-atom simulations of multiple plasma proteins, such as HSA, FG, IgG, and C3 randomly adsorbed onto cationic, anionic, and neutral PS nanoparticles, showing the structural change of adsorbed proteins and the formation of protein layers on the PS surface via the adsorption competition between proteins (Figure 9) [57]. In particular, we observed approximately twice higher diffusivities for proteins that are bound to either the particle surface or the boundary of protein layer than for those that are bound to both the particle surface and other proteins, indicating that the mobility of proteins depends on their positions in the protein layer, which helps to explain the experimental observations regarding the replacement of plasma proteins at the early stage of corona formation and the weaker binding in the outer protein layer than in the inner protein layer [57]. These all-atom and CG simulations have captured the replacement of plasma proteins on the nanoparticle surface and suggested the key factors controlling this competitive adsorption, which helps to explain the Vroman effect. However, only five or less proteins have been simulated in the solvent condition that is much simpler than the bloodstream, and the prediction of adsorption kinetics still needs to be improved, which will be achieved by developing force fields and multiscale methodologies.

## 4. Interactions between the Protein Corona-Particle Complex and Lipid Membrane

**Figure 10:**
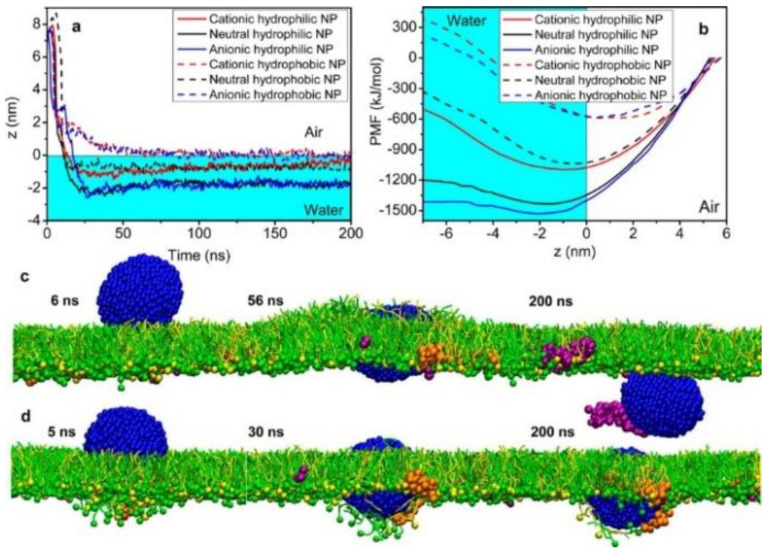
Calculations of PMFs for the interaction between NP and pulmonary surfactant monolayer (**a**,**b**) and snapshots from simulations of differently charged NPs interacting pulmonary surfactant proteins (SP-B1-25 and SP-C) and phospholipid monolayer (**c**,**d**). Reproduced with permission from [63], American Chemical Society, 2013.

Hu et al. performed CG simulations of protein-coated nanoparticles that were interacting with lipid monolayers, showing the effects of particle electrostatics and hydrophobicity on the adsorption of proteins onto the particle surface [63]. In particular, they found that protein adsorption influences the translocation of nanoparticles through a pulmonary lung surfactant (Figure 10) [63]. Ding and Ma’s CG simulations showed the adsorption of HSA into the nanoparticle surface, which promotes the binding between charged nanoparticles and membranes, but it also suppresses the insertion of hydrophobic nanoparticles into membranes, indicating that the effect of protein corona depends on the nanoparticle electrostatics and hydrophobicity [64].

**Figure 11:**
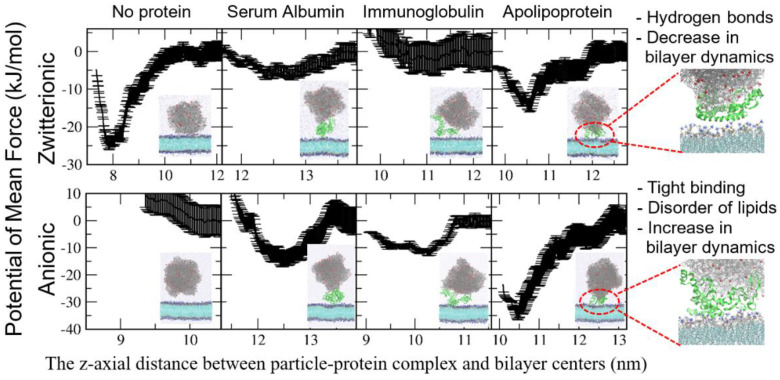
Free energy calculations of the 10 nm-sized PS particle complexed with plasma proteins adsorbed onto zwitterionic and anionic leaflets of lipid membrane. Reproduced with permission from [66], American Chemical Society, 2021.

Although these CG simulations have captured the effect of protein corona on the nanoparticle–membrane interaction, the results from these are not always easy to interpret at the atomic scale. To overcome this, all-atom simulations of the protein–nanoparticle complex and membrane have been performed. Duan et al. performed all-atom simulations of a BSA-adsorbed graphene interacting with lipid membrane, which showed that the BSA adsorption induces the increased graphene surface and specific electrostatic interactions between charged BSA residues and lipid headgroups, leading to the reduced extent of lipid extraction from membrane and the slower penetration of graphene into membrane [65]. Our group recently performed all-atom simulations of 10 nm-sized PS particles that were complexed with HSA, IgG, and APO proteins interacting with lipid bilayers and calculated their binding free energies, showing that the adsorbed proteins sterically weaken the interactions between nanoparticles and bilayers and, thus, suppress the nanoparticle–bilayer binding, which agrees well with experiments (Figure 11) [66]. However, this steric effect of adsorbed proteins occurs for the zwitterionic leaflet of bilayer, but not for the anionic leaflet of bilayer because of charge interactions between proteins and anionic lipid headgroups [66]. In particular, we found that proteins form hydrogen bonds with zwitterionic leaflets and, thus, restrict the lateral mobility of bilayers, as observed in experiments, while charge interactions between proteins and anionic leaflets disorder lipids and, thus, increase the lateral dynamics of bilayers [66].

## 5. Conclusions

The experimental and theoretical studies have revealed that protein corona influences conformations and surface properties of nanoparticles and their interactions with cell membranes, although those mechanisms have not been well understood at the atomic scale. As computer power and force-field development have advanced, MD simulations have provided considerable useful information regarding the formation of protein corona and its effect on nanoparticle properties and the nanoparticle-membrane binding for the past decade. In particular, MD simulations have been able to explore the competitive adsorption and desorption of plasma proteins on differently sized and charged nanoparticles, which helps to explain the experimental results and the Vroman effect.

Although MD simulations have captured the experimental observations of corona formation and its interactions with nanoparticles and membranes, there are still differences in environments and mass transport conditions of experiments and simulations, which precludes any quantitative comparison between the two. For instance, there are hundreds of plasma proteins that compete with each other and form protein corona on nanoparticle surfaces. Additionally, the flowing velocity of bloodstream may modulate protein–protein and protein–nanoparticle interactions. Cell membranes consist of various membrane proteins and lipids, and their components and ratios vary in different cells. Filaments of cytoskeleton influence the membrane shape and mechanical resistance and, thus, may modulate the protein-membrane interaction. These details should be considered to predict the adsorption kinetics and final composition of protein corona for drug delivery applications, which are expected to be resolved in the future by recent efforts in developing multiscale–simulation methodologies and realistic cell–membrane models. Despite these limitations, MD simulations have successfully interpreted the Vroman effect and experimental observations at the atomic scale, clearly indicating a promising tool for the rational design of highly efficient drug delivery systems.
